# Retrospective Analysis of Esophageal Heat Transfer for Active Temperature Management in Post-cardiac Arrest, Refractory Fever, and Burn Patients

**DOI:** 10.1093/milmed/usx207

**Published:** 2018-04-03

**Authors:** Melissa Naiman, Andrej Markota, Ahmed Hegazy, John Dingley, Erik Kulstad

**Affiliations:** 1 Collaborative for Advanced Research, Design, and Evaluation, University of Illinois at Chicago, 2121W. Taylor Street #540, Chicago, IL 60612.; 2 Medical Intensive Care Unit, University Medical Center Maribor, Ljubljanska 5, 2000 Maribor, Slovenia; 3 Department of Anesthesia & Perioperative Medicine, University Hospital, Rm. C3-108, London, ON, Canada N6A 5A5; 4 Welsh Centre for Burns, ABM University Health Board, Morriston Hospital, Swansea SA6 6NL, UK; 5 Department of Emergency Medicine, University of Texas Southwestern Medical Center, 5323 Harry Hines Blvd, Dallas, TX 75390.

**Keywords:** targeted temperature management, therapeutic hypothermia, critical care, burns, refractory fever, post-cardiac arrest

## Abstract

Core temperature management is an important aspect of critical care; preventing unintentional hypothermia, reducing fever, and inducing therapeutic hypothermia when appropriate are each tied to positive health outcomes. The purpose of this study is to evaluate the performance of a new temperature management device that uses the esophageal environment to conduct heat transfer. De-identified patient data were aggregated from three clinical sites where an esophageal heat transfer device (EHTD) was used to provide temperature management. The device was evaluated against temperature management guidelines and best practice recommendations, including performance during induction, maintenance, and cessation of therapy. Across all active cooling protocols, the average time-to-target was 2.37 h and the average maintenance phase was 22.4 h. Patients spent 94.9% of the maintenance phase within ±1.0°C and 67.2% within ±0.5°C (574 and 407 measurements, respectively, out of 605 total). For warming protocols, all of the patient temperature readings remained above 36°C throughout the surgical procedure (average 4.66 h). The esophageal heat transfer device met performance expectations across a range of temperature management applications in intensive care and burn units. Patients met and maintained temperature goals without any reported adverse events.

## Introduction

Temperature management is a standard of care across specialties; approximately 20 million cases each year in the United States warrant active temperature management. Preventing unintentional hypothermia, reducing fever, and inducing therapeutic hypothermia when appropriate are each tied to positive health outcomes and patients who develop uncontrolled hyperthermia or hypothermia experience worse outcomes. Patients who experience even mild cases of perioperative hypothermia (<1°C) lose ~16% more blood and are at ~22% increased relative risk for transfusion.^1^ Trauma patients admitted with a core temperature reading of <35°C were associated with a significantly increased mortality at 24 hr and 28 d (OR 2.72 [1.18–6.29] and OR 2.82 [1.83–4.35]), respectively.^2^ Cardiac arrest patients who develop fever following targeted temperature management experience increased mortality at 30 d and worsened neurological outcomes at 1 yr.^3^ In cases of severe traumatic brain injury, induced normothermia lowers mean intracranial pressure and lowers the total amount of time patients exhibit intracranial pressure over 25 mmHg.^4^ Targeted temperature management is strongly recommended in post-resuscitation guidelines issued by the American Heart Association^5^ and European Resuscitation Council,^6^ among other professional societies. Most recently, the World Health Organization recommended actively warming surgical patients to prevent surgical site infections.^7^

Despite extensive clinical evidence, active temperature management is either underutilized^8^ or inadequately applied^9^ in critical care populations. Both scenarios may be partially attributed to clinical challenges associated with the advanced devices used to control patient temperature. It is well established that advanced temperature management methods are more efficient than *ad hoc* methods, such as chilled saline bolus or ice packs. Advanced methods incorporate servo-controlled temperature monitoring, which allows a temperature management protocol to be automated and meet the patient’s exact needs. Currently, the most common commercially available devices use one of three mechanisms to modulate patient core temperature: intravascular convection, surface convection, or surface conduction. Intravascular devices rely on the surgical placement of a specialized central venous catheter; this method is effective, but must be placed by a trained physician under sterile conditions and requires access to a major blood vessel. Once placed, patients face an increased risk of Central Line Associated Blood Stream Infection^10^ and thrombosis.^11^ Surface devices that conduct heat through the skin obstruct patient access require that skin must be mostly intact and can cause skin damage and infection.^12,13^ There is no evidence to support that one technique is better than the other,^14^ so current recommendations support the use of any active temperature management method that a clinician is comfortable with and makes sense in their operational setting.^5^

**Figure 1.**
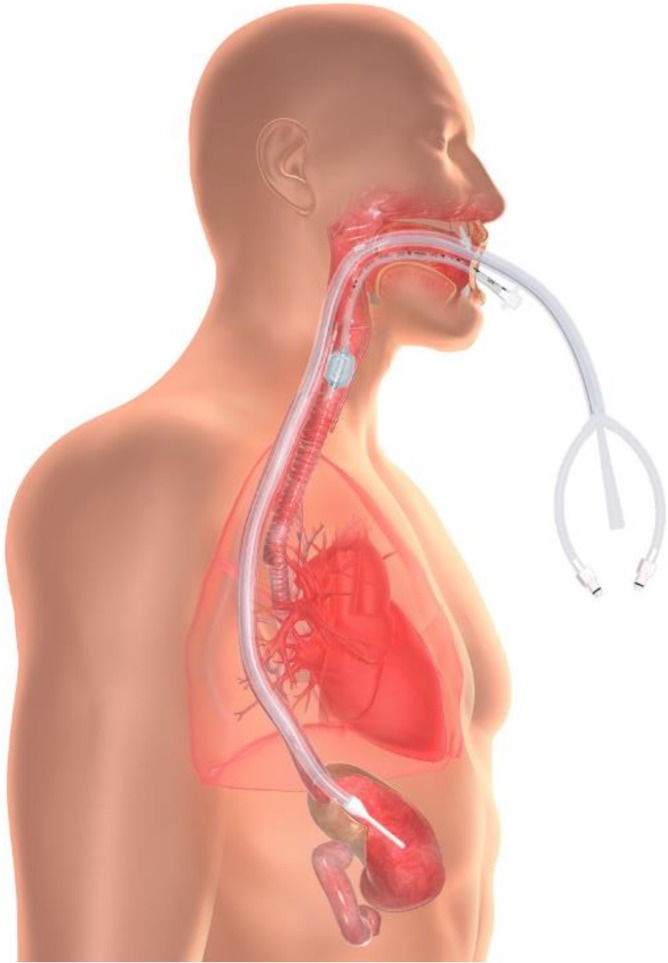
The EHTD is placed into the esophagus to conduct heat transfer. The device connects to standard suction (central lumen) and a standard water blanket chiller (two outer lumens) to circulate temperature-controlled water within the device.

Considering these risks, a new temperature management device was developed^15^ that performs heat transfer in the esophageal environment (Fig. 1). The device is constructed from medical grade silicone and connects to commercially available heat exchange units (commonly available in acute care settings) that circulate temperature-controlled water within the device (i.e., water never enters the GI tract, in contrast to lavage techniques). Any YSI 400 compatible temperature probe can be used to automate temperature monitoring. The risk profile and patient management requirements are similar to a standard OG tube. The device may be damaged by jaw clenching or chewing, so bite block placement is recommended. Prolonged contact with oral mucosa can result in minor lesions, so moving the tube regularly and/or using a securement device, such as a endotracheal tube holder, is strongly recommended. Patients receiving endoscopy following esophageal heat transfer have presented with esophageal lesions, but these were consistent with OG tube placement and not attributable to the device itself.^16^ The purpose of this study is to evaluate the performance of an esophageal heat transfer device (EHTD) in a variety of critical care scenarios.

## Evaluating Active Temperature Management Devices

Currently, there is not a “gold standard” for temperature management protocols, which creates some challenges when evaluating new temperature management devices. However, there is emerging clinical research and published best practices that can provide guidance for technology assessment. In the critical care environment, temperature management protocols (whether the goal is to reduce or increase core temperature) are described in three phases: induction, maintenance, and cessation of therapy.

Most modern studies measure induction in terms of time-to-target, defined as the time from the initiation of therapy until the goal temperature is achieved.^17,18^ For cooling protocols, the clinical importance of this parameter is derived from the relationship between reduced temperature and the downregulation or reduction of neurodegenerative biochemical processes, including mitochondrial dysfunction and apoptosis, glutamate release and free radical production, blood–brain barrier and cell membrane permeability, and brain metabolism.^19^ Resuscitation guidelines are the most explicit; the American Heart Association Guidelines and European Resuscitation Council Guidelines recommend patients reach target temperature within 4 hr after return of spontaneous circulation.^5,6^ There is insufficient evidence to support formal guidelines for time-to-target goals in patients experiencing forms of ischemia–reperfusion injury other than sudden cardiac arrest, but there is clinical evidence that fever (usually defined as body temperature above 38°C) aggravates inflammatory cascades that lead to worsened health outcomes;^20–22^ therefore, active fever prevention or reversal is a standard practice. When applying these guidelines and best practices to technology evaluation, to be clinically useful, an active temperature management device should demonstrate the ability to reach target temperature in under 4 hr.

The rationale for active warming is derived from another series of biochemical events linked to temperature, specifically coagulopathy and immune response.^1^ There is sufficient evidence for the World Health Organization to issue a clinical guideline^23^ that surgical patients should not experience a core temperature below 36°C at any point. Therefore, a relevant active temperature management device should maintain a temperature of at least 36°C as a procedure begins. In the case of severe burn patients, this may require the initiation of active warming preoperatively.

Once target temperature is achieved, the second phase of a temperature management protocol, most frequently termed “maintenance,” begins. For cooling protocols, the purpose of monitoring core temperature maintenance is to optimize neuroprotection. Poor neurological outcomes are associated with imprecise temperature maintenance,^14,24^ although there is insufficient evidence to support a clinical guideline for a deviation threshold. Therefore, the research community typically defines a “minor” deviation as greater than ± 0.5°C and deviations up to ±1.0°C are considered acceptable.^14,20,24^ In warming protocols, the goal is to maintain temperature above 36°C for the duration of the procedure, but how far above that threshold is left to clinicians’ discretion. In both scenarios, maintenance is typically reported as the proportion of time a patient remained within a defined range of the target. Therefore, to be clinically relevant, a temperature management device should be able to maintain a precise temperature from the time target is attained until the course of treatment is completed.

The final phase of a temperature management protocol is cessation of therapy. During cessation, patients must be monitored closely to ensure that discontinuing therapy is appropriate. In cooling protocols, and especially in post-cardiac arrest patients, rebound hyperthermia is a significant concern.^25^ To counteract this phenomenon, patients are rewarmed slowly (AHA guidelines recommend a rate of 0.25°C−0.5°C^5^) over a 12-h period. In neurogenic and central fever cases, temperature spikes can be unpredictable and may occur for several weeks.^26^ In warming protocols, temperature should not be permitted to drop below 36°C during recovery, although this metric is often not reported as part of perioperative temperature management studies. Therefore, to be clinically relevant, a temperature management device should demonstrate the ability to affect gradual temperature change without allowing rebound hyperthermia. It should also accommodate transitions across care sites (e.g., from the operating room to recovery) and resumption of therapy with minimal risk to the patient.

## Methods

De-identified data from patients treated with active temperature management using an EHTD (Catalog No. EnsoETM ECD01 or ECD02; Attune Medical, Chicago, IL, USA) were aggregated from three clinical sites for retrospective analysis. Patient demographics were reviewed to confirm consistency with device instructions for use (i.e., patient mass between 40 kg and 200 kg). Records were also reviewed for completeness to confirm that core temperature readings for each patient were recorded hourly; if measurements were recorded more frequently, temperature over an hour span was averaged. In all cases, EHTDs were connected to a Blanketrol III (Cincinnati Subzero, Cincinnati, OH, USA) or CritiCool (MTRE, Rehovot, Israel) commercially available heat exchange unit and core temperature measurements were obtained from a YSI 400 enabled Foley temperature probe placed in the bladder.

The evaluation criteria were established to mirror the three phases of temperature management protocols observed in critical care environment: induction, maintenance, and cessation of therapy (rewarming in post-cardiac arrest patients). Induction was evaluated by calculating the time-to-target, defined in this study as the time, in hours, from device placement to first temperature reading within ± 1°C of target. The maintenance phase was defined as the first measurement after target was achieved until therapy concluded (indicated by the initiation of a rewarming protocol in post-cardiac arrest patients or conclusion of therapy in all others). Maintenance stability was defined as the proportion of core temperature measurements within the target range during the maintenance phase. Rebound hypothermia was defined as any temperature readings above 38°C recorded after target temperature had been achieved in post-cardiac arrest protocols. Fever burden was defined as the average amount of time, in hours, a patient demonstrated temperature measurements above 38°C in fever reversal cases.

## Results

**Figure 2.**
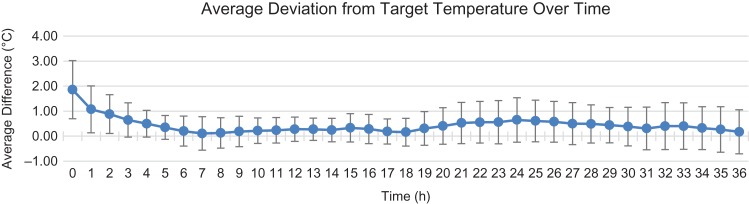
Average deviation (blue line) was calculated by subtracting the target temperature from the goal temperature and dividing by the total measurements recorded at a given time point. The error bars represent the standard deviation calculated for all measurements recording at a given time point. The increased variations in the first 4 hr are attributed to heterogenous starting temperatures across the patient population, especially among post-cardiac arrest cases with a 33°C target temperature. Increased variation toward the end of the protocol is consistent with unique patient response to rewarming, ranging from no excursions (typically if the patient suffered major neurological insult) to rebound hyperthermia.

A dataset of 1,000 core temperature readings was constructed from 30 patient records, representing 27 cooling protocols (23 post-cardiac arrest patients and 4 fever reversal patients) and 3 perioperative warming protocols for severe (~50% body surface area) burn patients. Across all protocols, the average deviation (calculated as actual temperature − target temperature/no. of readings at a time point, Fig. 2) from target at any time point was 0.43°C (SD = 0.72°C).

### Cooling Protocols

Across all active cooling protocols, the average time-to-target was 2.37 h (range 0–14 h, SD ± 2.8 h) and the average maintenance phase was 22.4 h (range 13–31 h). Patients spent 94.9% of the maintenance phase within ±1.0°C and 67.2% within ±0.5°C (574 and 407 measurements, respectively, out of 605 total).

**Figure 3.**
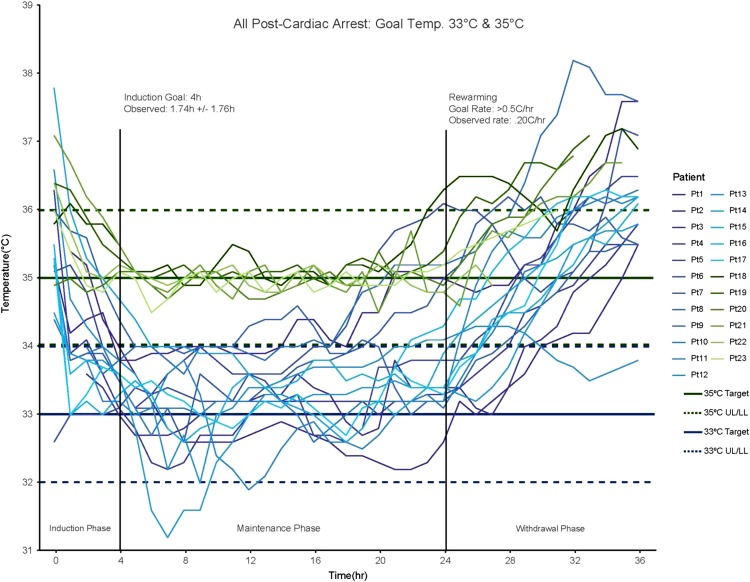
A total of 23 post-cardiac arrest, patients were treated with either a 33°C protocol (blues) or 35°C protocol (greens). The observed average time-to-target was 1.74 h and patients spent 94.9% of the maintenance phase within ±1.0°C and 67.2% within ±0.5°C (574 and 407 measurements, respectively, out of 605 total).

**Figure 4.**
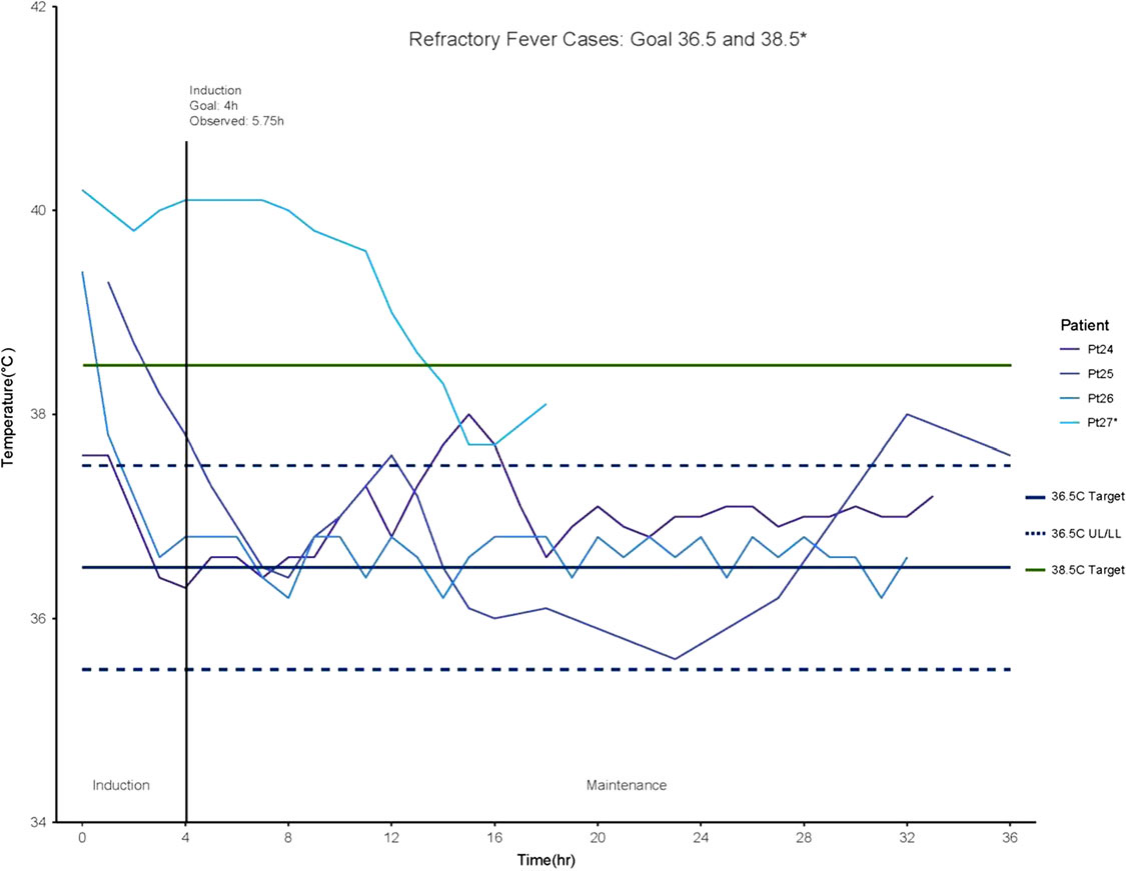
In four refractory fever cases, patients were treated with either a 36.5°C protocol or a 38.5°C protocol. The average time-to-target for fever reversal cases was 5.75 h (range 2–14 h) and average fever burden was 4.5 h (14.9% of total treatment time). Patient 27, the only recipient of a 38.5°C protocol, was in treatment for severe burns and developed refractory fever subsequent to skin graft surgery. The patient had been febrile for several hours before cooling was initiated and active cooling was withdrawn once the patient had maintained a core temperature below 38.5°C for five consecutive hours.

In post-cardiac arrest patients, average time-to-target was 1.78 h (range 0–6 h, SD ± 1.67 h), the average maintenance phase was 22.4 h (range 19–24 h). The average rewarming rate was 0.20°C/h and only one patient demonstrated fever rebound, with readings at 38.1°C–38.2°C for a total of 2 h (Fig. 3). The average time-to-target for fever reversal cases was 5.75 h (range 2–14 h) and average fever burden was 4.5 h (14.9% of total treatment time, Fig. 4). One case represented a significant deviation from other cooling protocols. Patient 27 was in treatment for severe burns and developed refractory fever subsequent to skin graft surgery.^27^ In this case, the patient had been febrile for several hours before cooling was initiated and active cooling was concluded once the patient had maintained a core temperature below 38.5°C for five consecutive hours, with a total fever burden of 13 h (72.2% of total treatment time). The other three cases were refractory fever with infectious origins who were actively cooled for between 32 h and 36 h with an average fever burden of 1.66 h (7.14% of total treatment time).

### Warming Protocols

**Figure 5.**
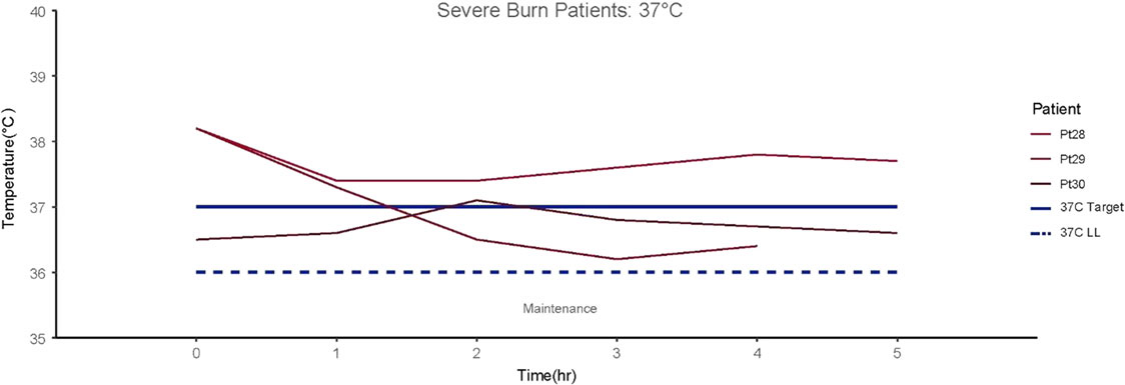
A warming protocol was conducted on three patients receiving skin graft surgery for severe burns. All patient temperature readings remained above 36°C throughout the surgical procedure (average 4.66 h).

In the warming cases, patients were warmed before entering the operating room and the objective was to avoid perioperative hypothermia while reducing the ambient temperature from 30°C to ~26°C.^27^ All of the patient temperature readings remained above 36°C throughout the surgical procedure (average 4.66 h, Fig. 5).

## Discussion

Based on the evaluation criteria established in this study, the EHTD could perform a variety of temperature management protocols. The average induction time of 2.37 h was well within the cardiac arrest guidelines and refractory fever recommendations of 4 h from the initiation of therapy until target was achieved. The EHTD also demonstrated stable temperature maintenance, patients who received a cooling protocol spent 94.9% of the maintenance phase within ± 1.0°C, and patients who received a warming protocol avoided inadvertent perioperative hypothermia for 100% of the surgical time. Cessation for cardiac arrest patients was controlled (rewarming rate of 0.20°C/h) and instance of rebound hypothermia was not significant.

This analysis supports EHTD effectiveness in real-world clinical settings; however, the dataset has some limitations. First, the dataset represented a very heterogenous sample in terms of many factors that affect temperature management, such as body composition, age, and comorbidities (details of many of the cases can be found elsewhere in the literature).^28–33^ Second, mild hypothermia protocols dominated the dataset (76% overall, and 56% received a 33°C target protocol) and were a potential source of bias. Finally, as this analysis is based on the data collected in hospital, it is impossible to account for the influence of any pre-hospital interventions, such as administering chilled saline. However, such interventions would be most influential on time-to-target, but unlikely to influence maintenance stability or post-hypothermia fever.

Despite these limitations, this evaluation has important future implications to temperature management in military medicine. First, the EHTD offers a new and effective temperature management technique that could be applied in forward critical care environments. Esophageal heat transfer offers several logistical advantages over surface and intravascular devices. The EHTD does not require extensive personnel to initiate treatment. Intravascular devices often require up to 30–45 min of hands-on physician and nursing time to set up and place, using sterile technique. Surface devices often require up to 15–20 min of hands-on time (and extensive patient manipulation) from two to three nurses. The EHTD can be placed in under 5 min by any provider trained to place an OG tube. Further, the EHTD is platform agnostic. Current surface and intravascular devices are designed only to connect within their own brand. The EHTD is designed to connect with the three most popular water blanket heat exchangers and evaluations are ongoing to expand approvals to other commercially available heat exchangers. This facilitates continuity of care during evacuation or transfers.

The EHTD can also be used in many severe burn cases. Clinicians treating burn patients often avoid surface and intravascular warming methods because any additional infection risk is considered unacceptable. Before the introduction of the EHTD, if surface and intravascular devices were excluded, the only remaining option available to counter perioperative hypothermia was elevating operating room ambient temperature as much as possible, sometimes up to 110 °F (43.3°C). However, raising room temperature is highly inefficient and transfers, at most, 13.2 W of energy to the patient^35^ while creating an extremely uncomfortable environment for the surgical team. In contrast, esophageal heat transfer delivers up to 30 W of energy to the patient, regardless of operating room temperature.^35^ In practice, during major burn excisions, anesthesiologists have used the EHTD to maintain normothermia in severe burn patients for up to 6 h in an OR kept at ~80 °F (26°C).^33^

## Conclusions

The data presented in this evaluation support that the EHTD meets the requirements for optimal temperature management in a variety of critical care patients. In addition to quantitative effectiveness measures, the device also offers several clinical and logistical advantages that may be of importance to field medicine. Taken together, these characteristics provide an opportunity to move temperature management (an established standard of care) forward. Additional studies are in progress to demonstrate the feasibility of the EHTD in other clinical environments. Research is underway to explore how anesthesia affects esophageal heat transfer, which will ultimately support clinical trials in inadvertent perioperative hypothermia prevention during procedures with substantial temperature management challenges, such as trauma surgery, spine surgery, and open abdominal/thoracic surgery. EHTD performance in cooling procedures will be further evaluated through data collected in a neurointensive care unit registry, focusing on subarachnoid and intracranial hemorrhage patients. Each of these studies will contribute to the overarching body of knowledge that will ultimately lead to expanded evidence-based temperature management guidelines that reach beyond resuscitation.

## Presentations

Presented as an oral presentation at the 2016 Military Health System Research Symposium (abstract number: MHSRS-16–1054).
